# Mother and Infant Body Mass Index, Breast Milk Leptin and Their Serum Leptin Values

**DOI:** 10.3390/nu8060383

**Published:** 2016-06-21

**Authors:** Francesco Savino, Allegra Sardo, Lorenza Rossi, Stefania Benetti, Andrea Savino, Leandra Silvestro

**Affiliations:** 1 Department of Pediatrics, Regina Margherita Children’ Hospital, Città della Salute e della Scienza di Torino, Piazza Polonia 94, Torino I-10126, Italy; allegra.sardo@gmail.com (A.S.); lory_rossi@hotmail.it (L.R.); stefy_benetti@virgilio.it (S.B.); andrea.savino817@edu.unito.it (A.S.); 2 Department of Public Health and Pediatric Sciences, University of Turin I-10126, Italy; leandra.silvestro@unito.it

**Keywords:** mothers, serum leptin, BMI, breast milk, infancy

## Abstract

Purpose: This study investigates correlations between mother and infant Body Mass Index (BMI), their serum leptin values and breast milk leptin concentration in early infancy. Subjects and Methods: We determined serum leptin values in 58 healthy infants and leptin values in their mothers’ breast milk, using radioimmunoassay (RIA). Infant and maternal anthropometrics were measured. Results: Median leptin concentration was 3.9 ng/mL (interquartile range (IQR): 2.75) in infant serum, 4.27 ng/mL (IQR: 5.62) in maternal serum and 0.89 ng/mL (IQR: 1.32) in breast milk. Median maternal BMI and weight were 24 kg/m^2^ (IQR: 4.41) and 64 kg (IQR: 15). Median infant BMI was 15.80 kg/cm^2^ (IQR: 4.02), while average weight was 5.130 kg (IQR: 1.627). Infants serum leptin values positively correlated with infants’ BMI (*p* = 0.001; *r* = 0.213) and breast milk leptin (*p* = 0.03; *r* = 0.285). Maternal serum leptin values positively correlated with maternal BMI (*p* = 0.000, *r* = 0.449) and breast milk leptin ones (*p* = 0.026; *r* = 0.322). Conclusion: Breast milk leptin and maternal BMI could influence infant serum leptin values. Further studies are needed to better elucidate the role of genetics and environment on infant leptin production and risk of obesity later in life.

## 1. Introduction

Leptin is a polypeptide hormone, made of 167 amino acids and discovered by Zhang *et al*. in 1994 thanks to studies on ob/ob gene in mice [1]. This hormone is the product of the ob gene, located on chromosome 7q31.3. It circulates in plasma free or bound to proteins and it exerts its action through the soluble-OB (s-OB) receptor [2]. The primary function of this hormone is to inhibit food intake and to promote energy expenditure by regulating neuronal activity in hypothalamic arcuate nuclei: leptin, in fact, activates anorectic Pro-opiomelanocortin/ Cocaine-and amphetamine-regulated-transcript (POMC/CART) neurons and hinders the activity of those which stimulate food intake Neuropeptide Y/Agouti-related protein (NPY/AgRP) [3].

There are increasing data that environmental factors in early life predict later health. The early adiposity rebound recorded in most obese subjects suggests that factors promoting body fat development operate in the first years of life [4]. It has been shown that higher serum leptin values correlate with lower body mass index (BMI) in childhood and with lower predisposition to develop metabolic disorders in adolescence and adulthood [5].

Schuster *et al*. suggest that milk leptin could provide a link between maternal body composition and infant growth and development and also plays a role in regulating infant appetite and food intake during early infancy [6].

Increased maternal body mass index is a well-established risk factor for later infant obesity [7,8,9] and prevalence of obesity women is increasing worldwide [10]. Evidence suggests that human milk may decrease the transmission of obesity from mothers to their children, for example exerting its effects on early growth of the infant microbiome, as recently proposed by Lemas *et al*. [11].

It is known that leptin is mainly produced by white adipose tissue; this is the reason why serum leptin values directly correlate with body fat stores. During fasting or weight loss, leptin levels decrease, while during overeating, they increase [12]. Leptin is also released by the hypothalamus, pituitary gland, skeletal muscle, stomach, liver, placenta and mammary gland [13,14].

Leptin is found in breast milk and, interestingly, it is not only related with infants’ body fat mass, but also with that of their mothers [15]. It is produced by mammary epithelial cells and it is associated with fat globules. Studies conducted on mice have shown that this hormone is transferred from maternal blood to breast milk and that it is then transferred from milk to mice puppies’ bloodstream. Interestingly, the presence of leptin receptors has been found on gastric and intestinal epithelial cells of both humans and rats, suggesting that leptin may play a role in the regulation of GI functions [16]. It could be assumed that leptin taken by children with breast milk can directly pass into their bloodstream through gut since leptin receptor isoform has been found in brush border, basolateral membrane, and cytoplasm of enterocytes [17].

The amount of different adipokines in human breast milk is supposed to affect energy intake of the infant. [18] It is not well determined whether only leptin plays a causal role in early life fat deposition prevention since recently it has been reported that also sOB receptor values could have a part in the regulation of infant energy intake and infant growth and development [19].

The aim of this study is to measure leptin in mother and infant serum and in breast milk in order to look for correlations between mother and infant BMI, their serum leptin values and breast milk leptin concentration in early infancy.

## 2. Materials and Methods

### 2.1. Subjects

#### 2.1.1. Infants

We enrolled 58 AGA healthy term infants who were admitted to the Department of Pediatrics of the University of Turin, Regina Margherita Children’s Hospital, between June 2013 and July 2015. The infants underwent blood tests during routine outpatient examinations. The study protocol was approved by the local Ethical Committee at Ospedale Mauriziano—Ospedale Infantile Regina Margherita (Ethical approval code: 4698, Protocol Version 1.0., 23 May 2013)—S. Anna Torino, and infants’ parents gave their written consent.

Criteria for enrollment were as follows:

Age: children from 10 days of life to 6 months and 15 days of life;
Gestational age: from 38 to 40 weeks;
Birth characteristics: birth weight from 2500 g to 4500 g, APGAR equal or above 7 and absence of neonatal diseases;
Nutrition: infants were fed with breast milk and they had not been weaned;
Clinical condition: at the time of blood sampling, infants did not have acute diseases and were afebrile.

At the time of sampling, infants were exclusively breastfed and they had not received any complementary feeding.

#### 2.1.2. Mothers

Fifty-eight caucasian mothers belonging to a rural or urban setting were enrolled with their children. Regarding delivery, 19 mothers underwent a Caesarian section, while 39 had a spontaneous delivery. Criteria for enrollment were as follows:

Mothers who delivered infants at 38 to 40 weeks’ gestation;
Mothers who were planning to exclusively breastfeed;

Mothers who signed written informed consent. Eligibility criteria for mothers were no maternal medical complications, non-smoking mothers, normal response to a glucose tolerance test, no mastitis, no prescribed medication, no digestive disorders.

### 2.2. BMI Measurement

Anthropometric measures were collected by two trained medical doctors with high intra-observer and inter-observer reliability.

Infants were weighed with an electronic integrating scale (SECA, model 757, Vogel & Halke, Hamburg, Germany), were measured in length with a stat meter and BMI was calculated as the ratio of body weight (kg) to the square of length (m^2^). Mothers were weighed with a scale (Wunder, Italy), measured in height with a stat meter (Holtain Limited, Crymych, Dyfed, UK) and BMI was calculated as above.

### 2.3. Blood Sampling and Hormone Analysis

For the evaluation of leptin in serum, infants underwent four hours fasting before blood testing usually at 8.00 in the morning. The sample was stored in a refrigerator for 60 min and was then put in the refrigerated centrifuge at 4 °C at 4000 revolutions/min for 10 min. The serum obtained was divided into 2 test tubes and was stored in a freezer at −30 °C. The same procedure was carried out for mothers.

Hormone analysis was conducted with a commercially available radioimmunoassay (RIA) kit (LEP R-40, Multispecie-Leptin-RIA-Sensitive, Mediagnost, Reutlingen, Germany) with a sensitivity of 0.04 ng/mL (0.01 ng/mL with the procedure for increased sensitivity). The intra-assay variation was less than 5%, and the inter-assay variation did not exceed 7.6%.

### 2.4. Breast Milk Sampling and Hormone Analysis

About 5 mL of foremilk samples were collected from the lactating women by hand expression between 07:00 and 09:00. All milk samples were collected in tubes containing protease inhibitors (Sigma-Aldrich Company Ltd., Dorset, England) and immediately frozen at −20 °C. Samples were thawed at 4–6 °C overnight and centrifuged at 2500 revolutions at 4 for 20 min to separate the fat milk. Like serum leptin, 2 mL of skimmed breast milk leptin was analyzed with a RIA kit (LEP R-40, Multispecie-Leptin-RIA-Sensitive, Mediagnost, Reutlingen, Germany) with a sensitivity of 0.04 ng/mL (0.01 ng/mL with the procedure for increased sensitivity).

### 2.5. Statistical Analysis

Statistical analyses were conducted using SPSS software (version 21.0, SPSS, Inc., Chicago, IL, USA). First, we performed univariate descriptive analysis. The normal distribution of the variables was tested by the Shapiro-Wilk test. Continuous variables were expressed as median and interquartile range (IQR). Data that were not normally distributed were analysed with the Mann-Whitney U test and the Kruskal-Wallis test. Correlations are expressed by the Spearman correlation coefficient. All tests were done with two tails, with a fixed significance alpha =5%.

## 3. Results

Median leptin concentration was 3.9 ng/mL (IQR: 2.75) in infant serum, 4.27 ng/mL (IQR: 5.62) in maternal serum and 0.89 ng/mL (IQR: 1.32) in breast milk (Table 1 and Table 2). Statistical significance was set at *p* < 0.05 and correlations were assessed using Spearman’s rho.

We evaluated the impact of potential confounders on breast milk leptin values and maternal and infant serum leptin values. Particularly, we analyzed the effect of infant age and gender on leptin concentrations.

Regarding infant age, we divided our cohort into three age groups at enrollment. We obtained a median (IQR) leptin concentration of 2.87 (2.53) ng/mL in infant serum, 3.27 (5.38) ng/mL in maternal serum and 0.83 (1.17) ng/mL in breast milk in group 1 (<2 months; *n* = 30), of 4.54 (9.89) ng/mL in infant serum, 2.46 (1.49) ng/mL in maternal serum and 1.18 (1.29) ng/mL in breast milk in group 2 (<4 months; *n* = 18) and of 4.85 (7.51) ng/mL in infant serum, 3.21 (2.25) ng/mL in maternal serum and 0.87 (3.55) ng/mL in breast milk in group 3 (4–6 months; *n* = 10). No significant differences in breast milk and infant and serum leptin values were detected among the three groups (*p* > 0.05).

We divided patients by gender into two groups: as concernes males (*n* = 26), the median (IQR) leptin concentration was 2.83 (2.16) ng/mL in infant serum, 3.27 (5.13) ng/mL in maternal serum and 0.83 (1.32) ng/mL in breast milk; in females (*n* = 32), the median (IQR) leptin concentration was 4.79 (8.46) ng/mL in infant serum, 2.84 (2.14) ng/mL in maternal serum and 0.93 (2.59) ng/mL in breast milk. With reference to gender, we did not observe any statistical differences in breast milk leptin values and maternal and infant serum leptin values (*p* > 0.05).

### 3.1. Infant Serum Leptin Values and Infant BMI

**Figure 1:**
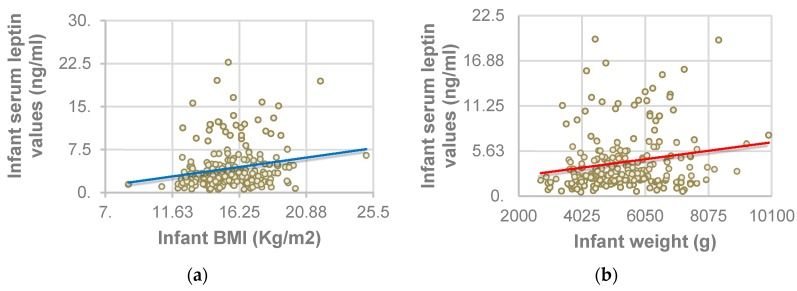
Correlation between infant serum leptin values and infant Body Mass Index (BMI) and weight. (**a**) Association between serum leptin values and BMI; (**b**) Association between serum leptin values and weight.

Serum leptin values positively correlated with infants’ weight (*p* = 0.002; *r* = 0.2) and BMI (*p* = 0.001; *r* = 0.213), as shown in Figure 1.

The positive correlation between infant serum leptin values and both infant BMI and weight suggests that leptin concentrations are directly related to body fat stores. This hormone is primarily released by adipocytes in adipose white tissue [15]. This is the reason why infants with higher BMI have higher serum leptin values [20].

### 3.2. Maternal Serum Leptin Values, Maternal BMI and Breast Milk Leptin

**Figure 2:**
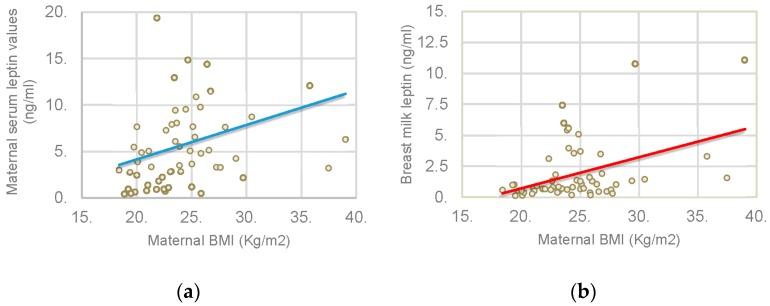
Correlation between maternal BMI and maternal serum leptin and breast milk leptin. (**a**) Association between maternal BMI and maternal serum leptin values; (**b**) Association between maternal BMI and breast milk leptin.

**Figure 3:**
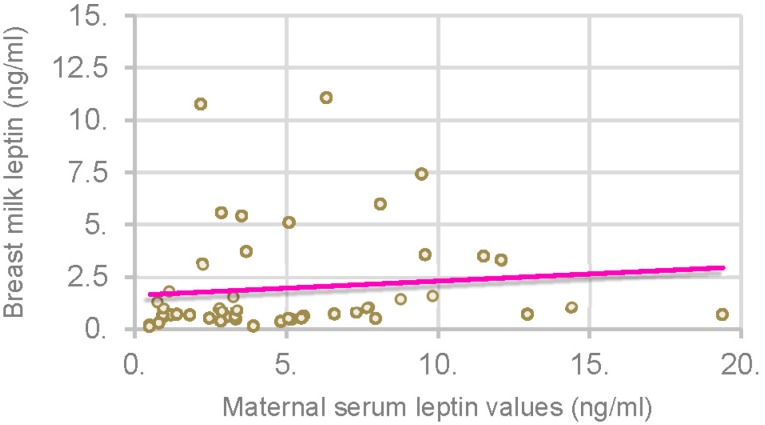
Correlation between maternal serum leptin and breast milk leptin.

Maternal BMI positively correlated with maternal serum leptin levels (*p* = 0.000; *r* = 0.449) and breast milk leptin (*p* = 0.004; *r* = 0.368) as illustrated in Figure 2. We found a significant correlation between breast milk leptin and maternal serum leptin values (*p* = 0.026; *r* = 0.322) as shown in Figure 3.

We found a positive and significant correlation between BMI and serum leptin values. As shown for infants, mothers with higher BMI have higher serum leptin values, suggesting that leptin concentration is directly proportional to body fat mass percentage [20].

Regarding breast milk, it is interesting that a positive correlation exists between maternal BMI and leptin levels in breast milk [21]. It could be that not only breast milk leptin depends on the amount produced by mammary epithelial cells, but also on the amount released from maternal body fat stores.

A significant correlation was observed between maternal serum leptin values and breast milk leptin [22]. Also Weyermann *et al*. [23] observed that leptin concentration in breast milk correlated positively with leptin in maternal serum.

### 3.3. Infant Serum Leptin Values, Maternal Serum Leptin Values and Breast Milk Leptin

**Figure 4:**
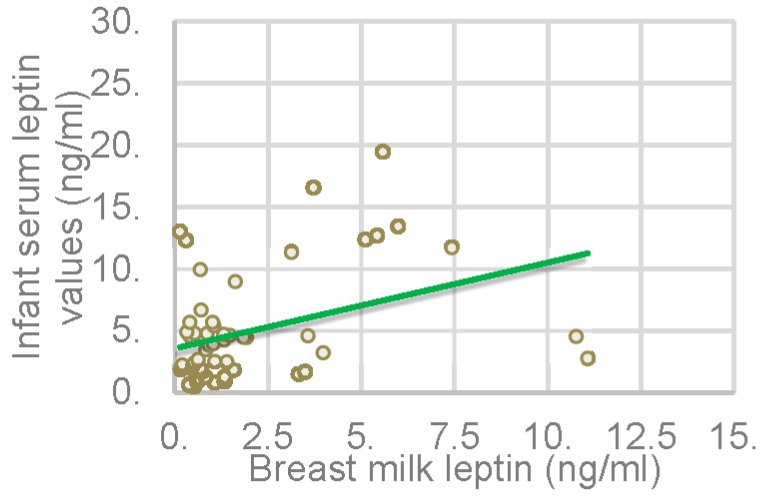
Correlation between breast milk leptin and infant serum leptin values.

We did not find any significant correlation between maternal and infant serum leptin values (*p* > 0.05), suggesting that further studies are required to investigate the possible role of maternal leptin in the regulation of infant metabolism [24]. Regarding breast milk leptin and infant serum leptin values, we obtained a positive correlation, as illustrated in Figure 4 (*p* = 0.03; *r* = 0.285).

The higher the breast milk leptin concentration is, the higher infant serum leptin values are. These findings suggest a possible association between breast milk components and infant adiposity [18].

## 4. Discussion

This study presents data of a positive correlation between breast milk leptin and infant serum leptin values. Furthermore, our study is strengthened by the fact that we found that breast milk leptin directly correlates with maternal serum leptin values. Actually, we did not obtain a significant association between maternal and infant serum leptin values. Regarding maternal and infant BMI, we showed that breast milk leptin and maternal serum leptin values directly correlate with materal BMI. In addition, we demonstrated a positive association between infant BMI and infant serum leptin values. We evaluated the possible impact of infant age and gender on infant and maternal serum leptin values and breast milk leptin concentrations. We did not obtain any significant differences in leptin values among the created groups.

### 4.1. Serum Leptin Values and BMI

Leptin is mainly produced by adipocytes; thus its levels are strictly associated to body fat mass percentage. During fasting, this hormone decreases; on the other hand, in overeating, its levels increase [25]. Both in infants and their mothers, we found that this hormone correlates with BMI and weight. Higher BMI correlates with higher serum leptin levels. It is known that people with an elevated BMI have high serum leptin levels not only because they have a larger amount of fat mass, but also because their adipocytes are bigger. What is more, Dusserre *et al*. showed that leptin values vary according to the type of adipose tissue that releases them: omental adipocytes express leptin mRNA less than subcutaneous adipocytes [26].

### 4.2. Breast Milk Leptin and Maternal Serum Leptin Values

Casabiell *et al*. showed that leptin is transferred from maternal bloodstream to breast milk in mice [16]. We found a positive correlation between breast milk leptin values and maternal serum leptin ones. It is thus possible that leptin in breast milk depends not only on the amount produced by mammary epithelial cells, but also on the amount in maternal bloodstream. It would be interesting to evaluate if maternal leptin values represent a predictor for infant obesity [27].

### 4.3. Breast Milk Leptin and Infant Serum Leptin Values

Leptin receptors have been found on gastrointestinal epithelial cells, suggesting that this hormone could be absorbed from infant mucosa and then transferred to infant bloodstream. The significant correlation that we found between breast milk leptin and infant serum leptin values could indicate that leptin in children is influenced by both infant fat stores and breast milk leptin. In previous studies, we demonstrated that formula fed infants have lower leptin levels than breast milk fed ones [28]. Data on the presence of leptin in infant formula are still controversial [29], however more investigations are needed to detect if hormones present in breast milk have a beneficial effect on obesity later in life [30,31].

### 4.4. Study Limitations

This study has several limitations. We could not assess the influence of leptin circadian variations since we do not have daily access to serum leptin samples, nor were we able to assess daily changes in breast milk leptin.

Moreover, we did not measure serum leptin at the same age time in all subjects enrolled.

However, baseline characteristics were similar among the infants in the study group.

Further, we were unable to measure fat mass at the same time of leptin sampling in mothers and infants.

Finally, since this study is observational it is important to interpret our correlations with caution.

Our findings are consistent with the possibility that breast milk leptin affects infant health later in life and opens new implications for research such as the role of breastfeeding and infant metabolic response.

Therefore, a follow-up of our patients, based on these results, will help us build a stronger overall evidence base and fill the gap in knowledge.

It is known that early nutrition plays an important role in the development of metabolic diseases in adolescence and adulthood. It has been shown that breastfed infants are at lower risk to become obese than formula-fed ones [32]. The positive correlation observed between maternal serum leptin concentration and maternal BMI is strictly linked to breast milk leptin values, suggesting that the amount of leptin in breast milk is influenced not only by mammary gland, but also by leptin released from maternal fat storages [33]. Interestingly, in a previous study, we demonstrated that infant serum leptin values are correlated to maternal BMI, thus showing that infants breast-fed by mothers with high BMI receive higher amounts of leptin [34]. Children with obese mothers seem to be at higher risk to become obese themselves [35]. The protective effect of breastfeeding against early childhood obesity may differ with race and ethnicity [36].

Many factors related to breastfeeding may influence childhood weight outcomes and obesity such as breastfeeding duration [37]; however, it should be considered that, ingesting high amounts of leptin, infants with obese mothers become leptin resistant and have alterations in appetite regulation [7,9,38]. In animal models it has been shown that obese phenotype can be transmitted by mothers to the following generations [39]. Since recently it has been observed that higher perinatal leptin is associated with lower adiposity at 3 years of life [40], leptin could be a key to understand the relationship between maternal BMI and infant growth and development. Intersting data showed that breast feeding affects infant’s self-regulation of milk intake during late infancy [41].

## 5. Conclusions

Understanding the determinants of infant body mass index is relevant for the study of childhood obesity and the related risk of obesity in adulthood.

The existing data of the effects of breast milk leptin on infant growth and adiposity are controversial. Growing evidence suggests that human milk may reduce the transfer of obesity from mother to progenies and leptin is one of the possible factors involved.

In this study we investigated the possible correlations between maternal and infant serum leptin values, breast milk leptin concentrations and infant and maternal BMI. We demonstrated a positive correlation between infants’ serum leptin concentrations and both maternal and infant BMI.

Regarding breast milk leptin values, we obtained a positive association not only with maternal BMI but also with maternal and infant serum leptin values.

There was no association between infant and maternal serum leptin concentrations.

Leptin is a peptide hormone produced by both adipocytes and mammary gland and it could be considered a marker that may signify excess fat accumulation.

Overall, our findings show that breast feeding and maternal BMI could influence infant serum leptin values. Further studies are needed to better elucidate the role of genetics on infant leptin production and risk of obesity later in life.

## Figures and Tables

**Table 1:** Infant anthropometric parameters and serum leptin values (median + interquartile range (IQR)).

Parameters	Infants
	*n* = 58
Age (days)	61 (76.5)
Gestational Age (weeks)	39 (1.5)
Birth Weight (kg)	3.275 (0.622)
Birth Length (cm)	49.45 (2.2)
Birth Cranial Circumference (cm)	34.05 (1.5)
Weight (kg)	5.130 (1.269)
Height (cm)	55 (3.25)
Cranial Circumference (cm)	39 (3)
BMI (kg/m^2^)	15.80 (2.47)
Serum Leptin (ng/mL)	3.9 (2.75)

**Table 2:** Maternal anthropometric parameters, serum leptin and Breast Milk (BM) leptin values (median + IQR).

Parameters	Mothers
	*n* = 58
Age (years)	28.5 (8)
Weight (kg)	64 (12.59)
Height (cm)	164 (0.064)
BMI (kg/m^2^)	24 (4.52)
Serum Leptin (ng/mL)	4.27 (5.62)
Breast Milk Leptin (ng/mL)	0.89 (1.32)

## References

[1] Zhang Y., Proenca R., Maffei M., Barone M., Leopold L., Friedman J.M. (1994). Positional cloning of the mouse obese gene and its human homologue. Nature.

[2] Tartaglia L., Dembski M., Weng X., Deng N., Culpepper J., Devos R., Richards G.J., Campfield L.A., Clark F.T., Deeds J. (1995). Identification and expression cloning of a leptin receptor. Cell.

[3] Spanswick D., Smith M., Groppi V.E., Logan S., Ashford M.L. (1997). Leptin inhibits hypothalamic neurons by activation of ATP-sensitive potassium channels. Nature.

[4] Rolland-Cachera M.F., Akrout M., Péneau S. (2012). Nutrient intakes in early life and risk of obesity. Int. J. Environ. Res. Public Health.

[5] Savino F., Liguori S., Benetti S., Sorrenti M., Fissore M.F., di Montezemolo L.C. (2013). High serum leptin levels in infancy can potentially predict obesity in childhood, especially in formula-fed infants. Acta Paediatr. Int. J. Paediatr..

[6] Miralles O., Sánchez J., Palou A., Picó C. (2006). A physiological role of breast milk leptin in body weight control in developing infants. Obesity.

[7] Andreas N., Hyde M., Gale C., Parkinson J., Jeffries S., Holmes E., Modi N. (2014). Effect of maternal body mass index on hormones in breast milk: A systematic review. PLoS ONE.

[8] Bider-Canfield Z., Martinez M.P., Wang X., Yu W., Bautista M.P., Brookey J., Page K.A., Buchanan T.A., Xiang A.H. (2016). Maternal obesity, gestational diabetes, breastfeeding and childhood overweight at age 2 years. Pediatr. Obes..

[9] Gruszfeld D., Kułaga Z., Wierzbicka A., Rzehak P., Grote V., Martin F., Poncelet P., Closa-Monasterolo R., Escribano J., Verduci E. (2016). Leptin and adiponectin serum levels from infancy to school age: Factors influencing tracking. Child Obes..

[10] NCD Risk Factor Collaboration (NCD-RisC) (2016). Trends in adult body-mass index in 200 countries from 1975 to 2014: A pooled analysis of 1698 population-based measurement studies with 19.2 million participants. Lancet.

[11] Lemas D.J., Young B.E., Baker P.R., Tomczik A.C., Soderborg T.K., Hernandez T.L. (2016). Alterations in human milk leptin and insulin are associated with early changes in the infant intestinal microbiome. Am. J. Clin. Nutr..

[12] Havel P., Townsend R. (1999). High-fat meals reduce 24-h circulating leptin concentrations in women. Diabetes.

[13] Hassink S., de Lancey E., Sheslow D.V., Smith-Kirwin S.M., O’Connor D.M., Considine R.V., Opentanova I., Dostal K., Spear M.L., Leef K. (1997). Placental leptin: An important new growth factor in intrauterine and neonatal development?. Pediatrics.

[14] Bado A., Levasseur S., Attoub S., Kermorgant S., Laigneau J.P., Bortoluzzi M.N., Moizo L., Lehy T., Guerre-Millo M., le Marchand-Brustel Y. (1998). The stomach is a source of leptin. Nature.

[15] Houseknecht K., McGuire M., Portocarrero C.P., McGuire M.A., Beerman K. (1997). Leptin is present in human milk and is related to maternal plasma leptin concentration and adiposity. Biochem. Biophys. Res. Commun..

[16] Casabiell X., Piñeiro V., Tomé María A., Peinó R., Diéguez C., Casanueva F. (1997). Presence of leptin in colostrum and/or breast milk from lactating mothers: A potential role in the regulation of neonatal food intake. J. Clin. Endocrinol. Metab..

[17] Barrenetxe J., Villaro C., Guembe L., Pascual I., Muñoz-Navas M., Barber A., Lostao M.P. (2002). Distribution of the long leptin receptor isoform in brush border, basolateral membrane, and cytoplasm of enterocytes. Gut.

[18] Fields D.A., Schneider C.R., Pavela G. (2016). A narrative review of the associations between six bioactive components in breast milk and infant adiposity. Obesity.

[19] Zepf F.D., Rao P., Moore J., Stewart R., Ladino Y.M., Hartmann B.T. (2016). Human breast milk and adipokines —A potential role for the soluble leptin receptor (sOb-R) in the regulation of infant energy intake and development. Med. Hypothesis.

[20] Sinha M., Caro J. (1998). Clinical aspects of leptin. Vitam. Horm..

[21] Uysal F., Onal E., Aral Y.Z., Adam B., Dilmen U., Ardiçolu Y. (2002). Breast milk leptin: Its relationship to maternal and infant adiposity. Clin. Nutr..

[22] Schuster S., Hechler C., Gebauer C., Kiess W., Kratzsch J. (2011). Leptin in maternal serum and breast milk: Association with infants’ body weight gain in a longitudinal study over 6 months of lactation. Pediatr. Res..

[23] Weyermann M., Beermann C., Brenner H., Rothenbacher D. (2006). Adiponectin and leptin in maternal serum, cord blood, and breast milk. Clin. Chem..

[24] Castagno E., Liguori S.A., Viola S., Lupica M.M., Oggero R., Savino F. (2009). Serum leptin levels in breastfed infants in the first six months of life, in their mothers and in breast milk. Dig. Liver Dis..

[25] Ahima R., Prabakaran D., Mantzoros C., Qu D., Lowell B., Maratos-Flier E., Flier J.S. (1996). Role of leptin in the neuroendocrine response to fasting. Nature.

[26] Dusserre E., Moulin P., Vidal H. (2000). Differences in mRNA expression of the proteins secreted by the adipocytes in human subcutaneous and visceral adipose tissues. Biochim. Biophys..

[27] Misra V., Straughen J., Trudeau S. (2013). Maternal serum leptin during pregnancy and infant birth weight: The influence of maternal overweight and obesity. Obesity.

[28] Savino F., Liguori S.A., Petrucci E., Lupica M.M., Fissore M.F., Oggero R., Silvestro L. (2010). Evaluation of leptin in breast milk, lactating mothers and their infants. Eur. J. Clin. Nutr..

[29] Lage M., Baldelli R., Camiña J.P., Rodriguez-Garci J., Peñalva A., Dieguez C., Casanueva F.F. (2002). Presence of bovine leptin in edible commercial milk and infant formula. J. Endocrinol. Invest..

[30] Savino F., Fissore M., Liguori S.A., Oggero R. (2009). Can hormones contained in mothers’ milk account for the beneficial effect of breast-feeding on obesity in children?. Clin. Endocrinol..

[31] Savino F., Liguori S., Fissure M., Oggero R. (2009). Breast milk hormones and their protective effect on obesity. Int. J. Pediatr. Endocrinol..

[32] Oddy W. (2012). Infant feeding and obesity risk in the child. Breastfeed. Rev..

[33] Savino F., Sorrenti M., Bennetti S., Lupica M.M., Liguori S.A., Oggero R. (2012). Resistin and leptin in breast milk and infants in early life. Early Hum. Dev..

[34] Savino F., Liguori S.A., Oggero R., Silvestro L., Miniero R. (2006). Maternal BMI and serum leptin concentration of infants in the first year of life. Acta Paediatr..

[35] Parsons T., Power C., Manor O. (2001). Fetal and early life growth and body mass index from birth to early adulthood in 1958 British cohort: Longitudinal study. BMJ.

[36] Ehrenthal D.B., Wu P., Trabulsi J. (2016). Differences in the protective effect of exclusive breastfeeding on child overweight and obesity by mother’s race. Matern. Child Health J..

[37] Modrek S., Basu S., Harding M., White J.S., Bartick M.C., Rodriguez E., Rosenberg K.D. (2016). Does breastfeeding duration decrease child obesity? An instrumental variables analysis. Pediatr. Obes..

[38] Doneray H., Orbak Z., Yildiz L. (2009). The relationship between breast milk leptin and neonatal weight gain. Acta Paediatr..

[39] Wang H., Ji J., Yu Y., Wei X., Chai S., Liu D., Huang D., Li Q., Dong Z., Xiao X. (2015). Neonatal overfeeding in female mice predisposes the development of obesity in their male offspring via altered central leptin signalling. J. Neuroendocrinol..

[40] Boeke C.E., Mantzoros C.S., Hughes M.D., Rifas-Shiman S.L., Villamor E., Zera C.A., Gillman M.W. (2013). Differential associations of leptin with adiposity across early childhood. Obesity.

[41] Li R., Fein S.B., Grummer-Strawn L.M. (2010). Do infants fed from bottles lack self-regulation of milk intake compared with directly breastfed infants?. Pediatrics.

